# Effects of “Bacuri” Seed Butter (*Platonia insignis* Mart.), a Brazilian Amazon Fruit, on Oxidative Stress and Diabetes Mellitus-Related Parameters in STZ-Diabetic Rats

**DOI:** 10.3390/biology11040562

**Published:** 2022-04-07

**Authors:** Jéssica Vanessa dos Santos Lindoso, Salmon Rocha Alencar, Andressa Amorim dos Santos, Renato Sampaio Mello Neto, Ana Victória da Silva Mendes, Mariely Mendes Furtado, Maisa Gomes da Silva, Ana Karolinne da Silva Brito, Emanuelle Karine Frota Batista, Silvia de Araújo França Baêta, Paulo Humberto Moreira Nunes, Massimo Lucarini, Alessandra Durazzo, Daniel Dias Rufino Arcanjo, Maria do Carmo de Carvalho e Martins

**Affiliations:** 1 Departamento de Biofísica e Fisiologia, Universidade Federal do Piauí, Campus Ministro Petrônio Portella, Teresina 64049-550, Brazil; jessik_vanessa@hotmail.com (J.V.d.S.L.); salmonalencar@hotmail.com (S.R.A.); amorimandressa13@outlook.com (A.A.d.S.); renato.sampaio.mn@gmail.com (R.S.M.N.); victoriams18@hotmail.com (A.V.d.S.M.); marielymf@live.com (M.M.F.); gomesmaisa@outlook.com (M.G.d.S.); anakarolinnesb@hotmail.com (A.K.d.S.B.); phmnunes@ufpi.edu.br (P.H.M.N.); daniel.arcanjo@ufpi.edu.br (D.D.R.A.); 2 Departamento de Clínica e Cirurgia Veterinária, Universidade Federal do Piauí, Centro de Ciências Agrárias, Teresina 64049-550, Brazil; emanuellefrota@yahoo.com.br (E.K.F.B.); silviavet2010@gmail.com (S.d.A.F.B.); 3 CREA—Research Centre for Food and Nutrition, Via Ardeatina 546, 00178 Rome, Italy; massimo.lucarini@crea.gov.it (M.L.); alessandra.durazzo@crea.gov.it (A.D.)

**Keywords:** bacuri, Clusiaceae, diabetes mellitus, antioxidant activity, hepatoprotection

## Abstract

**Simple Summary:**

The abnormal glucose metabolism present in diabetes mellitus causes several complications in different metabolic pathways and different organs. Chronic hyperglycemia promotes an imbalance between ROS production and antioxidant defense, causing oxidative stress, which contributes to damage in the body. The properties of natural products in diabetes mellitus research have been investigated to assist in the treatment. In this study, the effects of 28 days of oral administration of bacuri seed butter (*Platonia insignis* Mart.) was investigated on blood glucose, HbA1c, and liver and kidney function, as well as antioxidant defense in streptozotocin-induced female rats. Bacuri seed butter presented a positive effect on glycemic control, evidenced by a decrease in the percentage of glycated hemoglobin. Interestingly, the treatment also promoted increased hepatic antioxidant defenses and reduced liver damage, demonstrating a hepatoprotective effect.

**Abstract:**

This study aimed to investigate the effects of oral administration of *Platonia insignis* Mart. (“bacuri”) seed butter (BSB) on oxidative stress and diabetes mellitus-related parameters in streptozotocin-induced (STZ) diabetic rats. Diabetes mellitus was induced in female Wistar rats (180–250 g) by the intraperitoneal administration of STZ (45 mg/kg, b.w). BSB (25, 50, and 100 mg/kg) was administered to animals for four weeks. The effect on weight gain, food intake, blood glucose, glycated hemoglobin, hepatic transaminases, plasma and liver TBARS and MPO activity, erythrocyte SOD activity, non-protein sulfhydryl groups (SH-NP), and histopathology of the liver tissue was investigated. BSB at the dose of 100 mg/kg had a positive effect on the reduction in glycated hemoglobin percentage and increased albumin concentration, as well as decreased ALT and AST levels and increased SH-NP liver levels in treated animals compared to normal control rats. Moreover, BSB had no effects on weight gain, food intake, and fasting glucose. Thus, the BSB presented marked properties in improvement of hepatic antioxidant defenses, which demonstrates BSB as a potential hepatoprotective agent in metabolic disorders.

## 1. Introduction

The *Platonia insignis* Mart. is a fruitful tree found in the Brazilian Eastern Amazon, in the state of Pará and Northern Brazil called “Bacurizeiro” [1,2]. It has wide possibilities for its use in the food and wood industries. Due to the organoleptic characteristics of the pulp, the fruits are widely used for making juices, ice cream, creams, sweets, jams, or even consumed *in natura* [3], in line with food applications [4]. The peel represents 64% to 70% of the fruit’s weight, followed by seeds, whose participation varies from 13% to 26%. Pulp is the component that presents itself in a smaller proportion, representing only about 10% to 18% of the weight of the fruit [5,6]. Therefore, this production chain generates a high percentage of waste, such as peel and seeds, which represent more than half of the total fruit mass, discarded without use. From the perspective of circular economy and biorefinery, the fruit wastes represent a valuable source of value-added compounds [7].

The *P. insignis* seeds can be considered a promising source of bioactive compounds for the design of new phytomedicines. Previous reports have identified two compounds with pharmacological properties: 1,3-distearoyl-2-oleoylglycerol (TG1) [8], and garcinielliptone FC (GFC), a polyisoprenilated acylphloroglucinol with in vitro and in vivo antioxidant, antileishmanial, and vasorelaxant properties [9,10,11,12]. The therapeutic use in folk medicine is based on the use of “bacuri seed butter” obtained from decoction of *P. insignis* seeds for the treatment of diarrhea, skin problems, earaches, spider and snake bites, rheumatism, and arthritis [13,14]. Moreover, previous studies have reported *P. insignis* seeds neuroprotective [15], anti-inflammatory [16], and hypocholesterolemic effects with decrease of atherogenic risk in a diet-induced hypercholesterolemia model in hamsters [17], as well as also being able to promote low acute toxicity and antileishmanial and immunomodulatory effects [18,19,20].

Many studies show that the increase in production of reactive oxygen species (ROS) and decreased antioxidant defense is caused by chronic hyperglycemia, which leads to oxidative stress [21], a condition that results when the critical balance between free radical generation and antioxidant defenses become unfavorable [22,23,24,25]. Over the years or decades, prolonged hyperglycemia present in diabetes mellitus promotes the development of extensive and irreversible organic lesions that affect the eyes, kidneys, nerves, and large and small vessels, as well as causing altered blood coagulation [26]. Several lines of evidence indicate that all these mechanisms are activated by a single event: mitochondrial overproduction of reactive oxygen species [27,28]. In this sense, antioxidants counteract with different mechanisms of action the adverse reactions of ROS, as well as oxidative stress status [29].

The use of natural products in diabetes mellitus (DM) research has been investigated [30,31]. Although a previous study from our research group has reported the promising effects to *P. insignis* seed butter on lipid and hepatic profiles [17], studies regarding properties of *P. insignis* seeds in hyperglycemia and glycated hemoglobin are scarce, and moreover, the lack of histopathological investigation and role of antioxidant activity and oxidative stress in DM need to be assessed and clarified. Thus, the investigation of possible health benefits of bacuri seeds would clarify its pharmacological effect on DM, as well as the prevention of DM-associated oxidative stress. This study would also provide a new application for *P. insignis* seeds, which are normally underused and discarded by the food industry. Therefore, it is pertinent and relevant to investigate the effects of *P. insignis* seed butter in an experimental model of DM in rats.

## 2. Materials and Methods

### 2.1. Animals

Female Wistar rats, 180–250 g, 8–12 weeks old, were obtained from the Animal House of the Federal University of Piauí and maintained under standard laboratory conditions at a temperature of 23 ± 2 °C and 12/12-h light/dark cycle throughout all the experiments. Feed and water were provided *ad libitum* to all the animals. The experiments were conducted according to ethical principles established for the Animal Experimentation of the National Council for Animal Experimentation Control and the current national legislation—Law 11.794, of 10.8.2008 and Law 9.605, of 12.22.98 [32,33]. The research was approved by the Animal Experimentation Ethics Committee of the Federal University of Piauí under protocol number 513/18.

### 2.2. The Seed Butter from “Bacuri” (Platonia insignis *Mart.*)

Bacuri seed butter (BSB) was purchased from Amazon Oil Inc. (Ananindeua, PA, Brazil). The seeds butter from *Platonia insignis* Mart. (“bacuri”) has been extensively studied by our research group. Concerning its chemical composition, bacuri seed butter contains about 64% saturated fatty acids, 34% monounsaturated and 2% polyunsaturated, mostly palmitic acid and oleic acid. A chromatographic profile of two partition fractions demonstrated the presence of xanthones, such as alpha- and gamma-mangostin [34]. Moreover, 1,3-distearoyl-2-oleoylglycerol (TG1) [8] and garcinielliptone FC (GFC) have been previously reported as bioactive compounds isolated from *P. insignis* seeds [9].

### 2.3. Experimental Diabetes Induction

**Figure 1:**
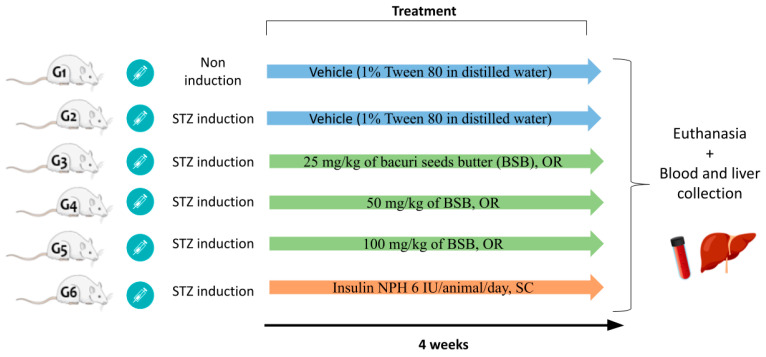
Graphical scheme of the experimental protocol of diabetes and BSB treatments.

The animals were divided into 6 groups with 5–8 animals each (Figure 1), and then fasted for 12 h and subsequently submitted to diabetes mellitus induction by a single intraperitoneal injection of streptozotocin (STZ) of 45 mg/kg (Cayman Chemicals, Ann Arbor, USA) dissolved in pH 4.5 citrate buffer [35].

In the first 24 h after induction, animals received 10% glucose solution to prevent hypoglycemia. Diabetes was confirmed by the elevated glucose levels in plasma, determined at 72 h. The animals with blood glucose greater than or equal to 250 mg/dL or 15 mM [36] were considered diabetic.

At the end of the experimental period (4 weeks), the animals were euthanized with an overdose of sodium thiopental (150 mg/kg, IP) preceded by lidocaine 10 mg/kg, IP. Immediately after euthanasia, blood and liver samples were collected for further analysis.

The doses of BSB used were based on previously performed studies on *Platonia insignis* Mart. biological effects [17]. The recommended insulin dose was used following the proposed by previous studies with similar DM model [37].

### 2.4. Biochemical Assays

Biochemical measurements of plasm glucose, urea, creatinine, AST, and ALT were performed using a Labmax Plenno^®^ automated biochemical analyzer and respective manufacturer kits (Labtest Inc., Lagoa Santa, MG, Brazil). Moreover, glycated hemoglobin (HbA1c) was determined by high-performance liquid chromatography (HPLC) in a blood sample.

Levels of thiobarbituric acid reactive substances (TBARS) and myeloperoxidase activity (MPO) were determined in plasma and liver homogenates [38,39]. Erythrocyte superoxide dismutase (SOD) and catalase (CAT) activities, as well as the liver concentration of non-protein sulfhydryl groups (SH-NP), were also performed [40,41].

### 2.5. Histopathology

Liver samples were prepared for histopathological evaluation by fixation in 10% formalin-buffered solution and subsequent sample cleavage. The fixed samples of tissue were sliced into small pieces of 3–5 mm and embedded in paraffin blocks. These blocks were sectioned using microtome. Sections were taken from paraffin blocks and stained with hematoxylin and eosin [42]. The stained slides were analyzed under a light microscope equipped with camera. Descriptive histological analysis was carried out by a trained examiner who was blind to the groups.

### 2.6. Statistical Analysis

Data were expressed as mean ± standard error of the mean (SEM). Statistical analysis was performed by one-way analysis of variance (ANOVA), followed by Tukey’s multiple comparisons test. The significance level was set at *p* < 0.05. For analysis of the results, the statistical program GraphPad Prism version 6.0 was used.

## 3. Results

The activity of the bacuri seed butter was explored as follows: (i) effect on body weight and food intake; (ii) effect on fast blood glucose levels and glycated hemoglobin (HbA1c) percentage; (iii) effect on biomarkers of liver and kidney function; (iv) effect on lipid peroxidation and myeloperoxidase activity in plasma and liver; (v) effect on antioxidant compounds; (vi) morphological effects on liver tissue.

### 3.1. Effect of Bacuri Seed Butter (Platonia insignis *Mart.*) Treatment on Body Weight and Food Intake

As shown in Table 1, diabetic animals showed weight loss and polyphagia, and the treatment with bacuri seed butter (BSB) did not interfere with these parameters. Differently, the group that received NPH insulin presented weight gain and lower daily feed intake.

### 3.2. Effects of Bacuri Seed Butter (BSB) (Platonia insignis *Mart.*) on Fast Blood Glucose Levels and Glycated Hemoglobin (HbA1c) Percentage

**Figure 2:**
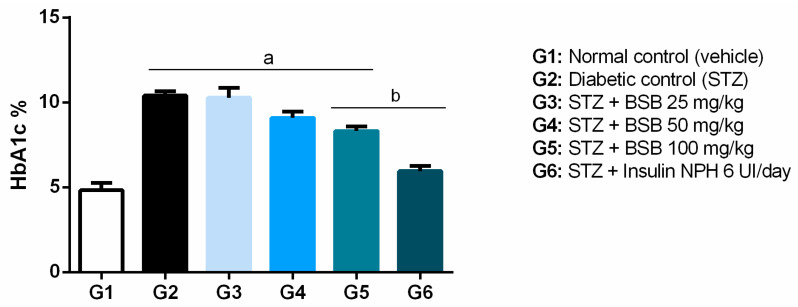
Percentages of glycated hemoglobin (HbA1C%) in STZ-induced diabetic rats treated with bacuri seed butter (BSB). ^a^ *p* < 0.05 vs. normal control; ^b^ *p* < 0,05 diabetic control; one-way ANOVA and Tukey’s post hoc test.

Regarding fasting blood glucose, treatment with BSB did not provide changes compared to diabetic control (Table 2). On the other hand, the group treated with 100 mg/kg BSB presented a reduction of the percentage of glycated hemoglobin, a long-term glycemic evaluation parameter, compared to the diabetic control (Figure 2).

### 3.3. Effects of Bacuri Seed Butter (BSB) (Platonia insignis *Mart.*) on Biomarkers of Liver and Kidney Function

Plasma concentrations of ALT, AST, and albumin, as well as relative liver weight were used as markers to evaluate liver function. The results are shown in Table 3. Diabetic animals presented higher ALT and AST levels and lower plasma albumin concentrations. Treatment with BSB at the three doses promoted a decrease in transaminase levels. It is noteworthy that the 100 mg/kg dose restored albumin concentration to normal levels. The insulin treatment also had a positive effect on reducing transaminases. Regarding the liver weight, all diabetic animals presented higher means compared to normal control.

Additionally, there was an increase of urea in diabetic control animals (106.1 ± 5.64 mg/dL) compared to normal control (34.88 ± 1.50 mg/dL), and the treatment with BSB did not change this parameter (G3: 102.6 ± 11.95 mg/dL; G4: 92.63 ± 6.3 mg/dL; G5: 111.8 ± 4.18 mg/dL), while insulin treatment promoted similar levels compared to the normal control group (30.60 ± 2.08 mg/dL). Creatinine levels had no changes between the experimental groups.

### 3.4. Effects of Bacuri Seed Butter (BSB) (Platonia insignis *Mart.*) on Lipid Peroxidation and Myeloperoxidase Activity in Plasma and Liver

**Figure 3:**
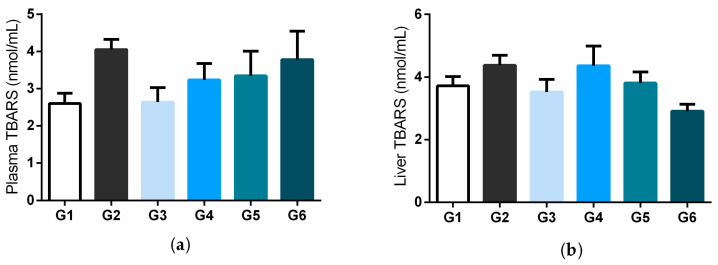
Plasma (**a**) and liver (**b**) concentrations of thiobarbituric acid reactive substances (TBARS) in STZ-induced diabetic rats treated with bacuri seed butter (BSB). Legend: normal control (G1); diabetic control (STZ) (G2); STZ + BSB 25 mg/kg (G3); STZ + BSB 50 mg/kg (G4); STZ + BSB 100 mg/kg (G5); STZ + insulin NPH 6 UI/day (G6).

Figure 3a,b illustrates plasma and liver concentrations for thiobarbituric acid reactive substances (TBARS), respectively. Plasma and liver levels had no significant differences between experimental groups.

**Figure 4:**
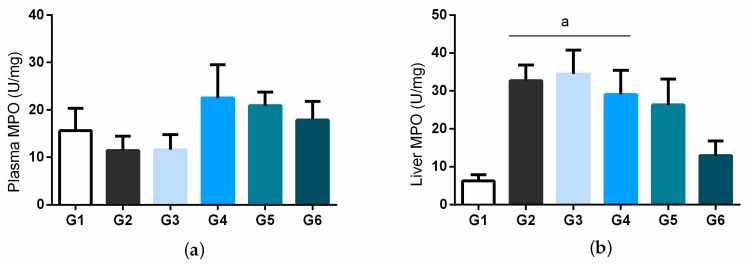
Plasma (**a**) and liver (**b**) concentration for myeloperoxidase (MPO) in STZ-induced diabetic rats treated with bacuri seed butter (BSB). Legend: normal control (G1); diabetic control (STZ) (G2); STZ + BSB 25 mg/kg (G3); STZ + BSB 50 mg/kg (G4); STZ + BSB 100 mg/kg (G5); STZ + insulin NPH 6 UI/day (G6). ^a^ *p* < 0.05 vs. normal control; one-way ANOVA and Tukey’s post hoc test.

Plasma myeloperoxidase (MPO) concentrations (Figure 4a) also did not differ significantly between groups. Figure 4B shows the liver activity of MPO, and it is possible to notice that the diabetic control group, diabetic treated with 25 mg/kg (BSB), and diabetic treated with 50 mg/kg (BSB) had increased levels of MPO compared to the normal. There was no statistical difference between the normal control and the group treated with the highest dose of bacuri seed butter (100 mg/kg), nor the group treated with insulin.

### 3.5. Effect of Bacuri Seed Butter (Platonia insignis *Mart.*) on Antioxidant Compounds

**Figure 5:**
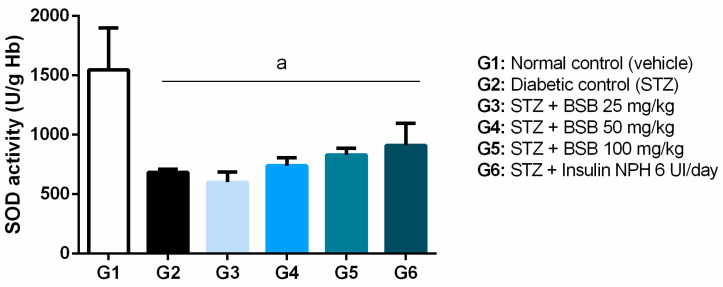
Antioxidant activity of superoxide dismutase (SOD) in erythrocytes of STZ-induced diabetic rats at the end of 4 weeks of treatment with bacuri seed butter (BSB). ^a^ *p* < 0.05 vs. normal control; one-way ANOVA and Tukey’s post hoc test.

**Figure 6:**
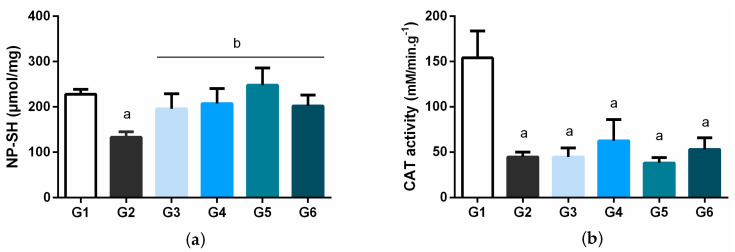
Hepatic concentrations of non-protein sulfhydryl groups (NP-SH) (**a**) and liver catalase (CAT) activity (**b**) in STZ-induced diabetic rats at the end of 4 weeks of treatment with bacuri seed butter (BSB). Legend: normal control (G1); diabetic control (STZ) (G2); STZ + BSB 25 mg/kg (G3); STZ + BSB 50 mg/kg (G4); STZ + BSB 100 mg/kg (G5); STZ + insulin NPH 6 UI/day (G6). ^a^ *p* < 0.05 vs. normal control; ^b^ *p* < 0.05 diabetic control; one-way ANOVA and Tukey’s post hoc test.

The activity of the antioxidant enzyme superoxide dismutase (SOD) in erythrocytes is presented in Figure 5. The SOD levels in the diabetic control group had a significant decrease (*p* < 0.05) compared to the normal control group. The treatment groups did not show a statistical difference compared to the diabetic control. Figure 6 shows the levels of non-protein sulfhydryl groups (SH-NP) and the catalase (CAT) activity in the liver homogenate of the animals. For both parameters, the diabetic control had significantly lower SH-NP levels and decreased CAT activity when compared to the normal group. Interestingly, all doses of the treatment with BSB restored liver SH-NP concentrations back to normal (Figure 6a). On the other hand, CAT activity was not restored after treatment with BSB (Figure 6b).

### 3.6. Morphological Changes in Liver Tissue of Diabetic Animals Treated with Bacuri Seed Butter (BSB) (Platonia insignis *Mart.*)

**Figure 7:**
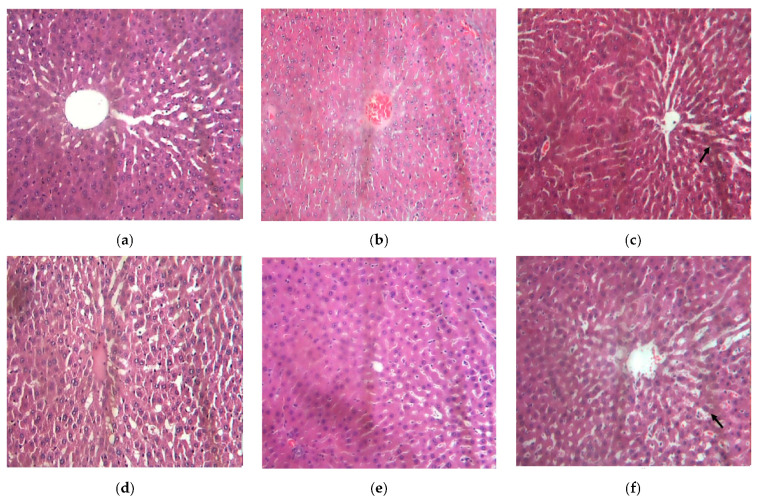
Representative photomicrograph of liver tissue from STZ-induced diabetic rats at the end of 4 weeks of treatment with bacuri seed butter (BSB). Legend: normal control (**a**); diabetic control (STZ) (**b**); STZ + BSB 25 mg/kg (**c**); STZ + BSB 50 mg/kg (**d**); STZ + BSB 100 mg/kg (**e**); STZ + insulin NPH 6 UI/day (**f**).

All liver tissue samples from the diabetic control group had moderate-to-mild multifocal hepatic congestion (Figure 7b), with some animals also showing hepatocyte hemorrhage, degeneration, and destruction (12.5%) compared to normal rats (G1) that revealed the absence of structural alterations in the hepatic parenchyma (Figure 7a). Liver tissue (100%) in animals treated with BSB at a dose of 25 mg/kg showed milder congestion (Figure 7c), which was more discrete compared to G2. The group treated with 50 mg/kg BSB had 75% of the animals with no structural alteration (Figure 7d), and 25% presented mild congestion. All analyzed liver samples from group G5 (100 mg/kg BSB) showed mild congestion (Figure 7e), and all samples from G6 (NPH insulin) showed cytoplasmic changes, with hepatocyte vacuolization (Figure 7f).

## 4. Discussion

Experimental models using diabetic rodents have been widely applied due to clinical, laboratory, and histopathological similarities with human diabetes [43,44,45]. Therefore, they are used to investigate possible pharmacological effects of different medicinal plants and bioactive compounds for the treatment of diabetes mellitus [35,46,47]. In the current study, the experimental model of streptozotocin-induced DM1 in Wistar rats was applied in order to assess the effects of bacuri seed butter (BSB) on oxidative stress and diabetic parameters after four weeks of oral treatment.

The chemical composition previously reported for bacuri seed butter retrieved garcinielliptone FC (GFC), a bioactive polyisoprenylated benzophenone, as one of the most pharmacologically studied compounds [9,10,11,12]. In general, polyisoprenylated benzophenones can be considered to be biomarkers widely identified with a peculiar prevalence in species from the Clusiaceae family, such as *P. insignis* [48]. Recent studies have reported for benzophenone-derivative compounds isolated from Clusiaceae species the inhibitory effect on alpha-amylase, which is open for prospections of polyisoprenylated benzophenones in the treatment metabolic disorders, since this property could provide benefits allied to antioxidant and anti-inflammatory effects [49,50]. Therefore, the *P. insignis* seed butter (BSB) as a source of GFC, as well as its effects on glycemia and oxidative stress related to diabetes mellitus, were investigated. The 4-week treatment with 100 mg/kg of BSB produced a decrease in the percentage of glycated hemoglobin (HbA1c) compared to the diabetic control group. Blood glucose fluctuates daily and, therefore, the HbA1c dosage (%) becomes a more reliable parameter of glycemic control, precisely because it has a positive correlation with the estimated mean blood glucose (EMBG), and this correlation is the fundamental basis for the use of HbA1c as a clinical parameter for diabetes control [27].

Although no fasting blood glucose-lowering effects were found with BSB treatment, the reduction in HbA1c in the 100 mg/kg BSB group indicated a hypoglycemic effect of this natural product in DM. Although capillary blood glucose is a simple and quick method of obtaining blood glucose, it measures only the current blood glucose, which can be influenced by several factors such as exposure to stress and is not useful for evaluating long-term glycemic control. Stress can increase plasma glucose and plasma corticosterone in rats [51]. In this way, even the stress of handling the animals can interfere with the result, making it more unstable and less reliable. A long-term and therefore more reliable measure of glycemic control is HbA1c. In this study, antioxidant effects of BSB may have contributed to the reduction of HbA1c.

The effects of BSB treatment on liver and kidney function biochemical markers were also evaluated to investigate other metabolic repercussions on DM and possible subchronic toxicity. One possible explanation for the elevation of liver cytolysis enzyme concentrations is related to hepatic toxicity of streptozotocin, causing liver damage. Supporting this hypothesis, the diabetic control group, treated only with the vehicle, presented high concentrations of ALT and AST, including with a higher mean than those found in the groups treated with BSB. Furthermore, STZ presents in addition to a diabetogenic effect, hepatotoxic and nephrotoxic action, as well as gastric ulceration [52,53].

Other studies have also found transaminases with high concentrations in STZ-induced diabetic animals [45,47,54]. These studies increase the evidence that the elevation of transaminases in the diabetic animal groups resulted from STZ toxicity and not from the effect of bacuri seed butter. It should be noted that treatment with BSB helped to reduce plasma transaminase concentration compared to diabetic control, suggesting that the treatment was effective in reducing liver damage.

Moreover, in a study already performed [18], the acute toxicity of BSB was evaluated, and the authors reported no death or behavioral change, so it was considered as a product with low acute toxicity. Considering the histopathological analysis of the liver, a hepatoprotective effect of BSB was identified, when compared with the presence of higher vascular congestion in the animals of the diabetic control group and mild or low congestion, or even absence of alterations in the animals treated with BSB. Moreover, the urea concentration and relative kidney weight of diabetic animals were higher than those found in the normal control group, indicating a possible toxic effect of STZ combined with protein metabolism disorder in DM. Related results have been reported in other studies [45].

Regarding the effect of BSB on oxidative stress and antioxidant defense markers, MPO activity in liver homogenate was higher in the diabetic control group animals compared to normal animals. On the other hand, the group treated with the 100 mg/kg dose of BSB and the insulin group presented values similar to those of the normal control group, which may indicate the presence of lower oxidative stress and inflammation in the liver, since MPO has been linked to inflammatory processes and the reactive species produced by activating transcription factors, such as NF-κB, expanding the inflammatory process [55,56]. It is noteworthy that bacuri seed butter is recognized for its anti-inflammatory potential [15,16,17].

The antioxidant defense was also investigated by the concentration of non-protein sulfhydryl groups (SH-NP) in liver homogenate. Diabetic control animals showed a decrease in SH-NP levels regarding the normal control, with another result proving the data that affirm that antioxidant defenses are decreased in DM [57,58]. It is noteworthy that groups receiving BSB treatment at the three doses (25, 50, and 100 mg/kg) presented higher levels of SH-NP, returning to similar levels compared to a normal control group. These data corroborate with some studies [17], wherein an increase in hepatic GSH levels was observed after acute treatment with BSB.

It is possible that the treatment time used in this study was insufficient to observe changes in this peroxidation marker, suggesting further studies with a longer time for hyperglycemia and treatment.

## 5. Conclusions

Bacuri seed butter (*Platonia insignis* Mart.) may have a positive effect on glycemic control, evidenced by a decrease in the percentage of glycated hemoglobin (100 mg/kg BSB). Furthermore, it promoted increased hepatic antioxidant defense, demonstrated by increased levels of SH-NP, as well as a potential hepatoprotective effect in this metabolic disorder, identified by the reduction in levels of biochemical markers of liver injury and lower damage signs in histopathology. Antioxidant and hepatoprotective effects of BSB represent a promising natural product for the management of diabetes alone or in combination with other therapies.

## Figures and Tables

**Table 1:** Effect of bacuri seed butter (*Platonia insignis* Mart.) on body weight and food intake of Wistar rats submitted to induction of diabetes mellitus.

	Normal Control	Diabetic Control	BSB			Insulin NPH 6 UI/Animal/Day
			25 mg/kg	50 mg/kg	100 mg/kg	
Body weight gain (g)	12.4 ± 2.5	−31.2 ± 4.8 ^a^	−31.6 ± 7.5 ^a^	−25.1 ± 3.1 ^a^	−35.6 ± 6.3 ^a^	28.4 ± 2.9 ^b^
Food Intake (g)	14.8 ± 0.4	35.9 ± 1.07 ^a^	32.4 ± 1.2 ^a^	31.7 ± 1.3 ^a^	32.9 ± 1.4 ^a^	22.2 ± 0.5 ^ab^

Means are reported as mean ± SEM. *p* < 0.05 was considered as statistically significant. ^a^ *p* < 0.05 vs. normal control; ^b^ *p* < 0.05 vs. diabetic control.

**Table 2:** Effect of bacuri seed butter on fasting blood glucose levels in STZ-induced diabetic rats after 4 weeks treatment.

Group	Fast Blood Glucose (mg/dL)			
	First Week	Second Week	Third Week	Fourth Week
Normal control	87.8 ± 4.3	99.6 ± 2.5	95.14 ± 4.1	91.43 ± 3.0
Diabetic control	477.0 ± 18.7 ^a^	515.0 ± 27.3 ^a^	515.4 ± 29.7 ^a^	539.7 ± 29.9 ^a^
BSB 25 mg/kg	509.4 ± 25.4 ^a^	514.6 ± 22.3 ^a^	497.8 ± 36.3 ^a^	470.2 ± 22.3 ^a^
BSB 50 mg/kg	468.8 ± 32.9 ^a^	543.2 ± 35.6 ^a^	600.0 ± 0.0 ^a^	554.2 ± 29.2 ^a^
BSB 100 mg/kg	486.4 ± 34.5 ^a^	497.8 ± 43.8 ^a^	532.0 ± 20.7 ^a^	571.2 ± 16.2 ^a^
NPH insulin	474.6 ± 20.4 ^a^	477.2 ± 19.4 ^a^	480.8 ± 30.1 ^a^	514.0 ± 31.8 ^a^

Means are reported as mean ± SEM. *p* < 0.05 was considered as statistically significant. ^a^ *p* < 0.05 vs. normal control.

**Table 3:** Liver function and injury markers in STZ-induced diabetic rats treated with bacuri seed butter (*Platonia insignis* Mart.).

	Normal Control	Diabetic Control	BSB			Insulin NPH 6 UI/Animal/Day
			25 mg/kg	50 mg/kg	100 mg/kg	
ALT (U/L)	47.00 ± 3.6	325.2 ± 26.9 ^a^	229.3 ± 17.3 ^ab^	195.3 ± 17.7 ^ab^	137.6 ± 21.6 ^ab^	62.0 ± 14.97 ^b^
AST (U/L)	111.1 ± 5.3	678.3 ± 64.6 ^a^	329.3 ± 59.1 ^ab^	287.5 ± 32.8 ^b^	192.6 ± 20.4 ^b^	161.2 ± 45.9 ^b^
Albumin (g/dL)	2.4 ± 0.1	1.77 ± 0.1 ^a^	1.88 ± 0.1 ^a^	1.8 ± 0.1 ^a^	2.2 ± 0.1 ^b^	2.1 ± 0.1
Liver relative weight (g)	2.9 ± 0.1	4.09 ± 0.1 ^a^	4.97 ± 0.2 ^a^	4.8 ± 0.1 ^a^	4.96 ± 0.1 ^a^	3.7 ± 0.2 ^ab^

Means are reported as mean ± SEM. *p* < 0.05 was considered as statistically significant. ^a^ *p* < 0.05 vs. normal control; ^b^ *p* < 0.05 vs. diabetic control.
